# Interleukin-10 contrasts inflammatory synaptopathy and central neurodegenerative damage in multiple sclerosis

**DOI:** 10.3389/fnmol.2024.1430080

**Published:** 2024-08-07

**Authors:** Luana Gilio, Diego Fresegna, Mario Stampanoni Bassi, Alessandra Musella, Francesca De Vito, Sara Balletta, Krizia Sanna, Silvia Caioli, Luigi Pavone, Giovanni Galifi, Ilaria Simonelli, Livia Guadalupi, Valentina Vanni, Fabio Buttari, Ettore Dolcetti, Antonio Bruno, Federica Azzolini, Angela Borrelli, Roberta Fantozzi, Annamaria Finardi, Roberto Furlan, Diego Centonze, Georgia Mandolesi

**Affiliations:** ^1^Neurology Unit, IRCCS Neuromed, Pozzilli, Italy; ^2^Faculty of Psychology, Uninettuno Telematic International University, Rome, Italy; ^3^Synaptic Immunopathology Lab, IRCCS San Raffaele Roma, Rome, Italy; ^4^Department of Systems Medicine, University of Rome Tor Vergata, Rome, Italy; ^5^Department of Human Sciences and Quality of Life Promotion, University of Rome San Raffaele, Roma, Italy; ^6^Clinical Trial Centre Isola Tiberina-Gemelli Isola, Rome, Italy; ^7^Department of Biomedicine and Prevention, University of Rome Tor Vergata, Rome, Italy; ^8^Clinical Neuroimmunology Unit, Institute of Experimental Neurology (INSpe), Division of Neuroscience, IRCCS San Raffaele Scientific Institute, Milan, Italy; ^9^IRCCS San Raffaele Scientific Institute, University Vita-Salute San Raffaele, Milan, Italy

**Keywords:** multiple sclerosis, interleukin-10, interleukin-1**β**, experimental autoimmune encephalomyelitis (EAE), GABA transmission, glutamate transmission

## Abstract

Proinflammatory cytokines are implicated in promoting neurodegeneration in multiple sclerosis (MS) by affecting excitatory and inhibitory transmission at central synapses. Conversely, the synaptic effects of anti-inflammatory molecules remain underexplored, despite their potential neuroprotective properties and their presence in the cerebrospinal fluid (CSF) of patients. In a study involving 184 newly diagnosed relapsing–remitting (RR)-MS patients, we investigated whether CSF levels of the anti-inflammatory interleukin (IL)-10 were linked to disease severity and neurodegeneration measures. Additionally, we examined IL-10 impact on synaptic transmission in striatal medium spiny neurons and its role in counteracting inflammatory synaptopathy induced by IL-1β in female C57BL/6 mice with experimental autoimmune encephalomyelitis (EAE). Our findings revealed a significant positive correlation between IL-10 CSF levels and changes in EDSS (Expanded Disability Status Scale) scores one year after MS diagnosis. Moreover, IL-10 levels in the CSF were positively correlated with volumes of specific subcortical brain structures, such as the nucleus caudate. In both MS patients’ CSF and EAE mice striatum, IL-10 and IL-1β expressions were upregulated, suggesting possible antagonistic effects of these cytokines. Notably, IL-10 exhibited the ability to decrease glutamate transmission, increase GABA transmission in the striatum, and reverse IL-1β-induced abnormal synaptic transmission in EAE. In conclusion, our data suggest that IL-10 exerts direct neuroprotective effects in MS patients by modulating both excitatory and inhibitory transmission and attenuating IL-1β-induced inflammatory synaptopathy. These findings underscore the potential therapeutic significance of IL-10 in mitigating neurodegeneration in MS.

## 1 Introduction

Increased levels of proinflammatory cytokines in the cerebrospinal fluid (CSF) of patients with multiple sclerosis (MS) have been associated with more severe prospective disease activity and disability (Rossi et al., 2015; Stampanoni Bassi et al., 2018; Magliozzi et al., 2020), and with higher degree of gray matter atrophy (Rossi et al., 2014; Magliozzi et al., 2018, 2020). Importantly, experimental studies in animal models of MS (experimental autoimmune encephalomyelitis, EAE) provided convincing evidence that a number of inflammatory mediators are directly involved in the neurodegenerative process that characterizes MS and EAE brains, unbalancing excitatory and inhibitory synaptic transmission and favoring excitotoxic neuronal damage (Centonze et al., 2009; Rossi et al., 2011a; Mandolesi et al., 2015). Notably, interleukin (IL)-1β, induces synaptic hyperexcitability and promotes excitotoxic neuronal damage by exacerbating glutamate-mediated neuronal excitability and reducing GABA-mediated inhibition in MS chimeric *ex-vivo* models (Rossi et al., 2012a,b; Mandolesi et al., 2015).

Anti-inflammatory mediators play key roles in the resolution of the inflammatory responses and may therefore exert beneficial effects on MS disease course. IL-10 is a major anti-inflammatory cytokine released by immune and glial cells, and is critically involved in the regulation of inflammatory activation in MS (Moore et al., 2001). Several studies indicate that IL-10 may provide a negative feedback signal during the inflammatory process (Goto et al., 1999; Moore et al., 2001; Ledeboer et al., 2002; Kettenmann et al., 2011; Kwilasz et al., 2015), and in fact, stimulation of IL-10 receptors reduces the activation of immune cells, the production of proinflammatory mediators, such as tumor necrosis factor (TNF), IL-1β, IL-6, IL-8, and promotes the expression of other anti-inflammatory molecules (Sabat, 2010; Porro et al., 2020). In line with the idea that IL-10 modulates acute inflammatory activity in MS, the expression of this molecule is reduced before relapses and increased during the remitting phases of the disease (Rieckmann et al., 1994; Waubant et al., 2001). Furthermore, low serum levels of IL-10 predicted the risk of relapse in patients with clinically isolated syndrome (Wei et al., 2019).

In various inflammatory and degenerative conditions, IL-10 has been associated with some neuroprotective effects (Porro et al., 2020), resulting from decreased production of inflammatory mediators, reduced microglial activation, and increased release of anti-inflammatory molecules and neurotrophins. In spite of its established role in contrasting the activation of immune cells, it is still unclear whether IL-10 may also regulate synaptic transmission, exerting a protective effect on synaptic damage induced by pro-inflammatory molecules.

Here, we evaluated in a group of relapsing remitting (RR)-MS patients the correlation between CSF levels of IL-10, brain atrophy and prospective disability. Moreover, in preclinical studies, we assessed whether IL-10 could influence excitatory and inhibitory synaptic transmission in EAE mice exerting protective effects on inflammatory neurodegeneration.

## 2 Materials and methods

### 2.1 RR-MS patients

We included 184 consecutive MS patients enrolled between 2018 and 2020. Patients were admitted to the Neurology Unit of IRCCS Neuromed (Pozzilli, Italy) and later diagnosed as suffering from RR-MS, based on clinical, laboratory and MRI parameters according to McDonald’s diagnostic criteria (Thompson et al., 2018). The study, performed in accordance with the Declaration of Helsinki, was approved by the Ethics Committee of IRCCS Neuromed (CE numbers 06/17). All patients gave written informed consent to participate in the study and to give their demographic and clinical data and biological material for scientific research. Inclusion criteria were the established RR-MS diagnosis and the ability to provide written informed consent to the study. Patients with different autoimmune and inflammatory disease or with clinically relevant medical or surgical conditions which might affect fatigue were excluded. At the diagnosis, demographic and clinical characteristics were recorded: age, sex, disease duration, radiological disease activity, clinical disability evaluated with the Expanded Disability Status Scale (EDSS) (Kurtzke, 1983) at the time of diagnosis and after 1 year follow-up. All patients had not received either corticosteroids or immunoactive therapies before lumbar puncture (LP). Disease modifying therapies (DMT) were initiated after diagnosis as follows: I line (interferon beta-1a 22 mcg = 7 patients; interferon beta-1a 44 mcg = 4 patients; teriflunomide = 12 patients; glatiramer acetate = 23 patients; dimethyl fumarate = 93 patients), II line (ocrelizumab = 7 patients; cladribine = 8 patients; fingolimod = 18 patients; natalizumab = 10 patients; rituximab = 2 patients).

### 2.2 CSF collection and analysis

In all patients, CSF was collected at diagnosis by LP. Oligoclonal bands (OCB) were assessed. After withdrawal, CSF was centrifuged and stored at −80°C. IL-10 was analyzed using a Bio-Plex multiplex cytokine assay (Bio-Rad Laboratories, Hercules, CA, United States). All samples were analyzed in triplicate. Concentrations were expressed as picograms/milliliter and a logarithmic transformation was applied.

### 2.3 MRI examination

Brain and spine MRI were performed at diagnosis. MRI included dual-echo proton density sequences, FLAIR, T1-weighted spin-echo (SE), T2-weighted fast SE, and contrast-enhanced T1-weighted SE after intravenous gadolinium (Gd) infusion (0.2 mL/kg), performed on 1.5 Tesla scanner. Radiological disease activity was defined as the presence of a gadolinium-enhancing (Gd+) lesion on MRI scans performed at diagnosis.

In a subgroup of 76 patients, cortical thickness, T2 lesion load and the volume of some subcortical structures (lateral ventricles, caudate, putamen, thalamus, globus pallidus, hippocampus, amygdala, accumbens) were extracted at time of diagnosis by using a 3 T MRI scanner (GE Signa HDxt, GE Healthcare, Milwaukee, WI, United States). The 3 T MRI scan included a 3D Spoiled Gradient Recalled (SPGR) T1-weighted sequence (178 contiguous sagittal slices, voxel size 1 × 1 × 1 mm^3^, TR 7 ms, TE 2.856 ms, inversion time 450 ms) and a 3D FLAIR sequence (208 contiguous sagittal 1.6 mm slices, voxel size, 0.8 × 0.8 × 0.8 mm^3^, TR 6000 ms, TE 139.45 ms, inversion time 1827 ms). We first segmented white matter lesions from FLAIR and T1 images by using the lesion growth algorithm as implemented in version 2.0.15 of the lesion segmentation tool (www.statisticalmodelling.de/lst.html (accessed on 14 May 2022)) for SPM12 (https://www.fl.ion.ucl.ac.uk/spm (accessed on 14 May 2022)). Furthermore, we used the computational anatomy toolbox (CAT12, version 916, https://dbm.neuro.uni-jena.de/cat/ (accessed on 14 May 2022)) as implemented in SPM12 to extract individual cortical thickness values from lesion filled MR images. T2 lesion load was computed from 3D T1 and 3d FLAIR images by using an online tool for white matter lesions segmentation (Coupé et al., 2018). Finally, the volume of some subcortical structures was extracted from 3D T1 MRI by using a well-established pipeline (Manjón and Coupé, 2016) (Supplementary Figure S1).

### 2.4 Experimental animals

Six-to eight-week-old female C57BL/6 mice (Charles-River, Italy) were randomly assigned to standard cages (4–5 animals per cage) and kept at standard housing conditions, including a Techniplast Mouse House^®^, with a light/dark cycle of 12 h and free access to food and water. All the efforts were made to minimize the number of animals utilized and their suffering. Animal experiments were carried out according to the Internal Institutional Review Committee, the European Directive 2010/63/EU and the European Recommendations 526/2007 and the Italian D. Lgs 26/2014.2.3.

### 2.5 EAE model

Chronic-progressive EAE was induced as previously described (Gilio et al., 2022). Eight–ten weeks old C57BL/6 female mice were active immunized with an emulsion of mouse myelin oligodendrocyte glycoprotein peptide_35–55_ (MOG35–55, 85% purity; Espikem, Prato, Italy) in Complete Freund’s Adjuvant (CFA; Difco, Los Altos, CA, United States), followed by intravenous administration of pertussis toxin (500 ng; Merck, Milan, Italy) on the day of immunization and two days post immunization (dpi). CFA mice received the same treatment as EAE mice without the MOG peptide, including complete CFA and Pertussis toxin. Animals were daily scored for clinical symptoms of EAE according to the following scale: 0 no clinical signs; 1 flaccid tail; 2 hindlimb weakness; 3 hindlimb paresis; 4 tetraparalysis; and 5 death due to EAE; intermediate clinical signs were scored by adding 0.5. For electrophysiological and molecular experiments animals were sacrificed during the acute phase of the disease (17–25 dpi).

### 2.6 Electrophysiology

Mice were killed by cervical dislocation, and corticostriatal coronal slices (190 μm) were prepared from fresh tissue blocks of the mouse brain using a vibratome. A single slice was transferred to a recording chamber and submerged in a continuously flowing artificial cerebrospinal fluid (ACSF) (34°C, 2–3 mL/min) gassed with 95% O2–5% CO2. ACSF composition was (in mM) 126 NaCl, 2.5 KCl, 1.2 MgCl_2_, 1.2 NaH_2_PO_4_, 2.4 CaCl_2_, 11 Glucose, and 25 NaHCO_3_. The striatum could be readily identified under low power magnification, whereas individual neurons were visualized *in situ* using a differential interference contrast (Nomarski) optical system. This employed an Olympus BX50WI (Japan) non inverted microscope with 40× water immersion objective combined with an infra-red filter, a monochrome CCD camera (COHU 4912), and a PC compatible system for analysis of images and contrast enhancement (WinVision 2000, Delta Sistem, Italy). Recording pipettes were advanced towards individual striatal cells in the slice under positive pressure and visual control (WinVision 2000, Delta Sistemi, Italy) and, on contact, tight GΩ seals were made by applying negative pressure. The membrane patch was then ruptured by suction and membrane current and potential monitored using an Axopatch 1D patch clamp amplifier (Molecular Devices, Foster City, CA, United States). Whole-cell access resistances measured in voltage clamp were in the range of 5–20 MΩ. Whole-cell patch clamp recordings were made with borosilicate glass pipettes (1.8 mm o.d.; 2–3 MΩ), in voltage-clamp mode, at the holding potential of −80 mV.

To study spontaneous glutamate-mediated excitatory postsynaptic currents (sEPSCs), the recording pipettes were filled with internal solution of the following composition (mM): K + -gluconate (125), NaCl (10), CaCl_2_ (1.0), MgCl_2_ (2.0), 1,2-bis (2-aminophenoxy) ethane-N,N,N,N-tetra acetic acid (BAPTA; 0.5), HEPES (19), GTP; (0.3), Mg-ATP; (1.0), adjusted to pH 7.3 with KOH. Bicuculline (10 μM) was added to the external solution to block GABAA-mediated transmission.

To study GABA-mediated spontaneous inhibitory postsynaptic currents (sIPSCs), the recording pipettes were filled with internal solution of the following composition (mM): 110 CsCl, 30 K + -gluconate, 1.1 EGTA, 10 HEPES, 0.1 CaCl_2_, 4 Mg-ATP, 0.3 Na-GTP. MK-801 (25 μM) and CNQX (10 μM) were added to the external solution to block, respectively, NMDA and non-NMDA glutamate receptors. For *ex vivo* experiments slices taken from control and EAE mice were perfused with IL-10 (Peprotech, 100 ng/mL) for 10 min.

Synaptic events were stored by using P-CLAMP 9 (Axon Instruments) and analyzed offline on a personal computer with Mini Analysis 6.0.3 (Synaptosoft, Leonia, NJ, United States) software. The detection threshold of both sEPSCs and sIPSCs was set at twice the baseline noise. The fact that no false events would be identified was confirmed by visual inspection for each experiment. Offline analysis was performed on spontaneous synaptic events recorded during fixed time epochs (1–2 min). Only data from putative GABAergic medium spiny neurons (MSNs) were included in the present study and identified immediately after rupture of the GΩ seal, by evaluating their firing response to the injecting of depolarizing current (typically tonic, with little or no adaptation). One to four cells per animal were recorded and three to five animals were sacrificed for each experimental condition.

### 2.7 RNA extraction and quantitative real-time PCR

CFA (*n* = 11) and EAE mice (*n* = 14) were sacrificed through cervical dislocation at 17–21 dpi to quickly remove both left and right striata. All the procedures were performed in RNAse-free conditions and samples were stored at −80°C until use. Total RNA was extracted according to the standard miRNeasy Micro kit protocol (Qiagen). Next, 400–500 ng of total RNA was reverse-transcribed using High-Capacity cDNA Reverse Transcription Kit (Applied Biosystem) according to the manufacturer’s instructions. About 20 ng of cDNA was amplified with SensiMix II Probe Kit (Bioline, Meridian Life Science) in triplicate using the Applied Biosystem 7900HT Fast Real Time PCR system to evaluate the expression of Il10 (Mm01288386_m1) and Il1b (Mm00434228_m1) mRNAs. Data were represented relative to CFA samples by using the ΔΔCt method and β-actin (Actb, Mm00607939_s1) was used as internal control for the normalization.

### 2.8 Statistical analysis

#### 2.8.1 RR-MS patients

Statistical analysis was performed with R. Continuous variables were reported in terms of median (first-third quartile). Categorical variables were presented as absolute frequency and percentage. To evaluate the association between IL-10 CSF levels and the volume of specific subcortical structures, nonparametric Spearman’s correlation was calculated. A linear regression model was performed to better explore the effect of the IL-10 on the variation of EDSS after a 1 year follow-up, adjusting for age at time of diagnosis, sex, disease duration, radiological activity, type of therapy (I or II line) and EDSS at time of diagnosis. Logarithmic transformation was applied to obtain a better approximation to the normal distribution. Spearman’s rho correlation was applied to evaluate association between covariates and variation of EDSS and Mann–Whitney to evaluate difference in sex, radiological activity and type of DMT. The significance level was established at *p* < 0.05.

#### 2.8.2 Preclinical experiments

Statistical analysis was performed with Prism GraphPad version 9.0. Data were presented as mean ± SEM. Throughout the text “*n*” refers to the number of cells recorded, except for molecular experiments, where it means the number of mice used. A paired student’s *t*-tests was used to compare two population means, before and after IL-10 incubation on the same cell. For comparisons between two groups and non-parametric values the Mann–Whitney test was performed. The association between mRNA levels of IL-10 and IL-1β were explored by nonparametric Spearman’s correlation. The significance level was established at *p* < 0.05.

## 3 Results

### 3.1 CSF levels of IL-10 negatively correlate with clinical disability measured 1 year after the diagnosis

**Figure 1:**
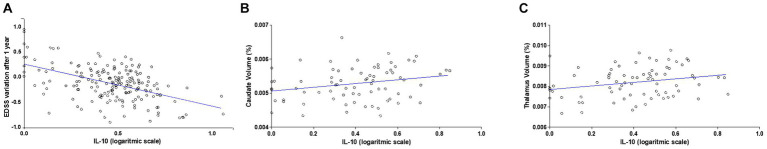
IL-10 levels in the CSF of RR-MS patients negatively correlate with disability and positively correlate with caudate and thalamic volumes. **(A)** Correlation plot shows a negative correlation between IL-10 CSF levels at the time of diagnosis and EDSS after 1 year follow-up. A negative correlation was observed (*N* = 184, Spearman’s rho = −0.19, *p* < 0.001). **(B)** Correlation plots show a positive correlation between CSF IL-10 levels, caudate volume (Spearman’s *r* = 0.231, *p* = 0.045) and **(C)** thalamic volume (Spearman’s *r* = 0.239, *p* = 0.037) at the time of diagnosis (*N* = 76).

We quantified the CSF levels of IL-10 in 184 RR-MS patients at the time of diagnosis (median = 0.02 pg./mL; IQR = 0–0.07). The clinical characteristics of MS patients are shown in Table 1. To assess the possible protective effect of IL-10 on MS disease course, we performed correlation analysis between CSF IL-10 levels and EDSS after 1 year of follow-up. We found a negative correlation between IL-10 and EDSS after 1 year (Spearman’s rho = −0.19, *p* = 0.008) and a corresponding negative correlation with EDSS variation 1 year after diagnosis (Spearman’s rho = −0.25, *p* < 0.001). To better explore the effect of IL-10 on the EDSS variation, a regression model was performed adjusting for age at diagnosis, sex, disease duration, radiological activity, EDSS at diagnosis, and DMT type (I vs. II line) (Supplementary Table S1). As shown in Figure 1A, CSF IL-10 levels (on logarithmic scale) strongly correlated with EDSS variation (beta = −0.76, SE = 0.23; *p* = 0.001).

**Table 1:** Demographic and clinical characteristics of MS patients.

RR-MS patients	*N*	184
Sex, F	*N* (%)	125 (67.9)
Age at diagnosis, years	Median (IQR)	34.6 (26.6–45.3)
Disease duration, months	Median (IQR)	5.7 (1.6–24.9)
OCB, presence	*N* (%)	144 (80)
Radiological disease activity, yes	*N* (%)	74 (40.2)
EDSS at diagnosis	Median (IQR)	1.5 (1–2)
DMTs (I/II line)	*N* (%)	139 (75.5)/45 (24.4)
EDSS after 1 year	Median (IQR)	1 (1–2)

Missing data: OCB 4/184 patients (2.2%).

No associations were found at the time of diagnosis between the CSF levels of IL-10 and clinical characteristics (age at diagnosis, sex, disease duration, radiological activity at diagnosis, EDSS at diagnosis).

### 3.2 IL-10 CSF levels positively correlates with the volumes of caudate and thalamus of RR-MS patients

To evaluate a possible neuroprotective effect of IL-10 circulating in the CSF of RR-MS patients at time of diagnosis, we investigated MRI structural measurements in a subgroup of 76 RR-MS patients. The volumes of specific subcortical structures (lateral ventricles, caudate, putamen, thalamus, globus pallidus, hippocampus, amygdala, accumbens) were analyzed and a significant positive correlation was found between IL-10 and both caudate (Spearman’s *r* = 0.231, *p* = 0.045; Figure 1B) and thalamic volumes (Spearman’s *r* = 0.239, *p* = 0.037; Figure 1C). These associations were maintained after data adjustment for age and disease duration (Caudate: Spearman’s rho = 0.27, *p* = 0.020; Thalamus: Spearman’s rho = 0.28, *p* = 0.018). No significant correlations were found with the other brain structures analyzed.

To better define a possible neuroprotective effect of IL-10 we explored its role in the inflammatory synaptopathic processes occurring in the striatum of the mouse MS model (EAE).

### 3.3 IL-10 levels directly correlate with IL-1β in the EAE striatum

**Figure 2:**
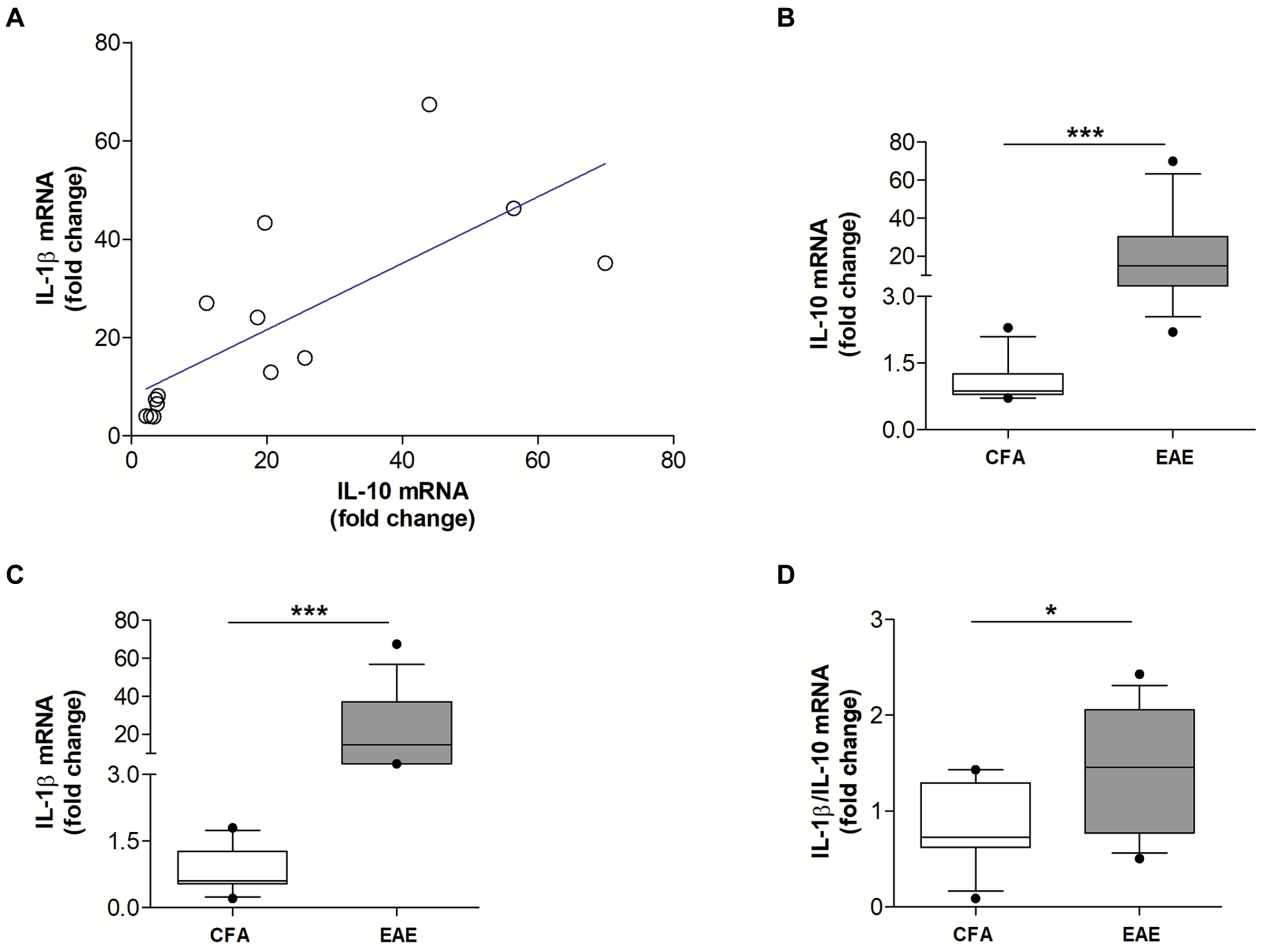
The mRNA levels of IL-10 and IL-1β positively correlate in the EAE striatum. **(A)** Correlation plot show a positive association between the mRNA levels of IL-10 and IL-1β in the EAE striatum (*n* = 14; Spearman’s *r* = 0.868, *p* < 0.001***). **(B,C)** Box-and-whisker plots show higher striatal mRNA levels of both IL-10 **(B)** and IL-1β **(C)** in EAE striatum relative to CFA group (EAE, *n* = 14; CFA, *n* = 11; Mann–Whitney U Test ***, *p* < 0.001). **(D)** Box-and-whisker plot show a higher IL-1β/IL-10 ratio in EAE condition relative to CFA group (Mann–Whitney U Test *, *p* < 0.05).

We previously demonstrated that the proinflammatory cytokine IL-1β is a key molecular player of synaptic damage seen in MS- and EAE-brains (Rossi et al., 2012a,b; Mandolesi et al., 2015). Since recent evidence from the literature showed that activation of IL-10 receptor leads to inhibition of IL-1β release (Sun et al., 2019), here we explored the possible antagonistic interaction between IL-1β and IL-10 by analyzing their expression in the EAE striatum. To this aim, MOG_35–55_ EAE was induced in a group of mice (*N* = 14) to perform qPCR quantification of both cytokines in the striatum at the peak for the disease. As a control group we used CFA mice (*N* = 11). As shown in Figure 2A, it emerged a direct correlation between mRNA levels of IL-10 and IL-1β (Spearman’s *r* = 0.868, *p* < 0.001) (Figure 2A), indicating that these two cytokines are co-expressed during neuroinflammation. The results also showed that mRNA levels of both IL-10 (*p* < 0.001; Figure 2B) and IL-1β (*p* < 0.001; Figure 2C) were significantly increased in EAE compared with control mice, in accordance with the neuroinflammatory processes occurring in EAE condition. Notably, the calculation of the ratio between IL-1β and IL-10 mRNA levels supported the prevalence of pro-inflammatory versus anti-inflammatory stimuli in the EAE striatum (*p* < 0.05; Figure 2D).

### 3.4 Direct effects of IL-10 on striatal neurotransmission in control mice

**Figure 3:**
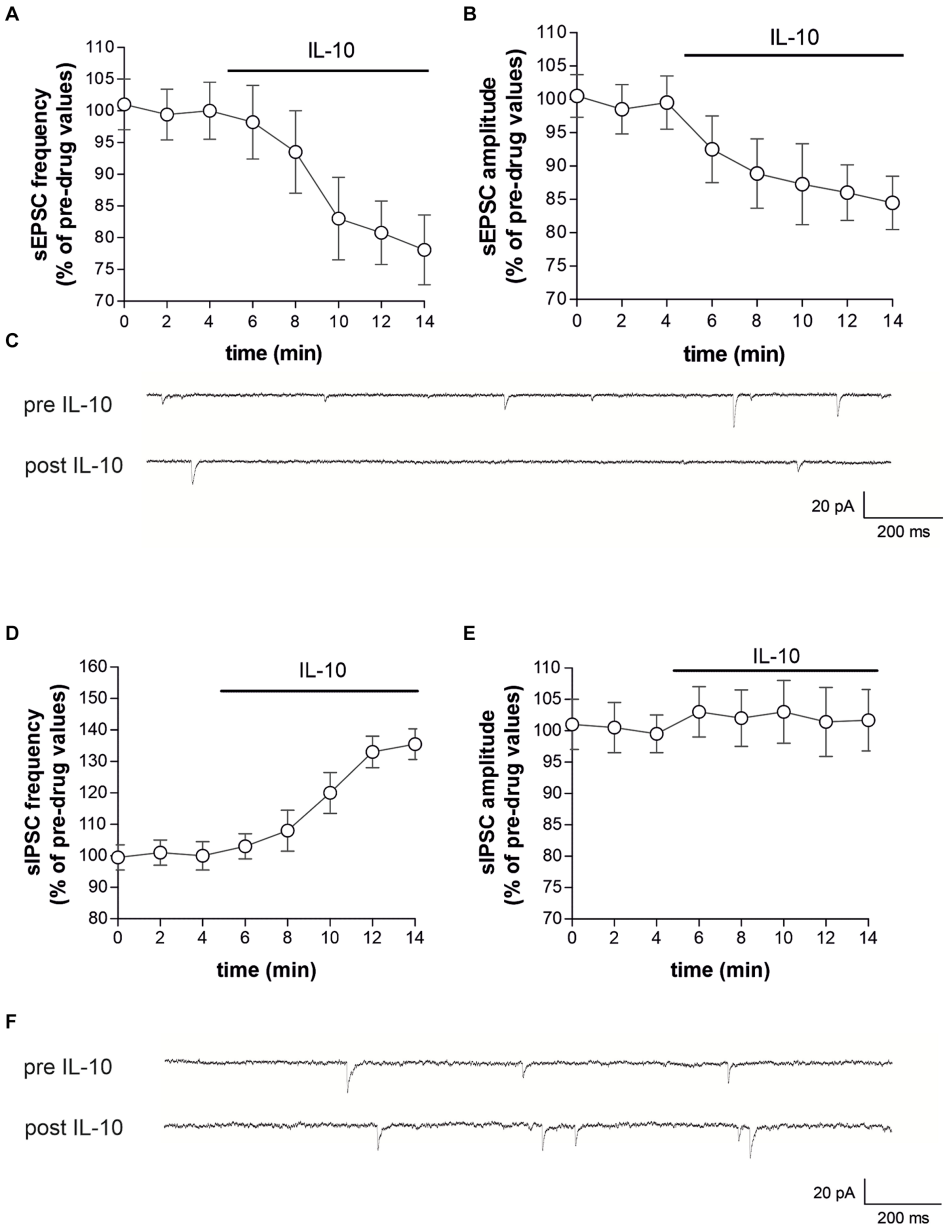
IL-10 reduces glutamatergic transmission and increases GABA transmission in control striatal slices. **(A–F)** Whole-cell patch-clamp recordings from MSNs of C57B/L6 corticostriatal slices incubated with IL-10 (100 ng/mL, 10 min). **(A–C)** The graphs show that IL-10 significantly reduced sEPSC frequency (A; paired Student’s *t*-test, *p* < 0.05 respect to pre-drug values), without affecting the amplitude (B; paired Student’s *t*-test *p* > 0.05). **(C)** Representative electrophysiological traces of the sEPSCs before and after ten minutes of IL-10. **(D–F)** The graphs show that application of IL-10 significantly increased the sIPSC frequency (D; Paired Student’s *t*-test *p* < 0.01 respect to pre-drug values), without affecting GABA amplitude (D; Paired Student’s *t*-test *p* > 0.05 respect to pre-drug values). **(F)** Representative electrophysiological traces of the sIPSCs before and after 10 minutes of IL-10. IL, interleukin; MSNs, medium spiny neurons; sEPSCs, spontaneous glutamate-mediated excitatory postsynaptic currents; sIPSCs, spontaneous GABA-mediated inhibitory postsynaptic currents.

To characterize whether IL-10 could affect synaptic function and possibly contrast the synaptic action of IL-1β, we first tested the effect of this molecule on the spontaneous synaptic transmission in the striatum of C57/BL6 mice. In particular, we recorded the sEPSCs (Figures 3A–C) and the sIPSCs (Figures 3D–F) from MSNs of corticostriatal slices before and after perfusion with IL-10 (100 ng/mL, 7–10 min). As shown in Figure 3, the sEPSC frequency was significantly reduced after IL-10 incubation (Paired *t*-test, *p < 0.05 respect to pre-drug values; *n* = 8; pre-IL-10 = 2.63 ± 0.24–post-IL-10 = 2.00 ± 0.14; Figures 3A,C; Supplementary Figures S2A,B), while the sEPSC amplitude was unaffected (Paired *t*-test, *p* > 0.05; *n* = 8; pre-IL-10 = 13, 62 ± 0.97–post-IL-10 = 12.20 ± 1.01; Figures 3B,C; Supplementary Figure S2). Conversely, the sIPSC frequency was significantly enhanced after IL-10 delivery (Paired *t*-test, **p < 0.01 respect to pre-drug values; *n* = 8; pre-IL-10 = 1.67 ± 0.26–post-IL-10 = 2.22 ± 0.39; Figures 3D,F; Supplementary Figures S2C,D). As observed for glutamate events, the sIPSC amplitude was unaffected by IL-10 (Paired *t*-test, *p* > 0.05; *n* = 8; Figures 3E,F), suggesting a presynaptic effect of IL-10 on both inhibitory and excitatory transmission.

Furthermore, we did not observe differences on sEPSC kinetic parameters after perfusion with IL-10 (data not shown; rise: pre-IL-10 = 0.91 ± 0.04–post-IL-10 = 0.98 ± 0.13; decay: pre-IL 10 = 5.16 ± 0.36–post-IL-10 = 5.42 ± 0.44; h-w: pre-IL-10 = 6.29 ± 0.43–post-IL-10 = 6.99 ± 0.88; Paired *t*-test: *p* > 0.05), indicating that IL-10 has no effect on excitatory postsynaptic activity.

### 3.5 IL-10 exerts a neuroprotective effect on striatal neurotransmission in EAE mice

**Figure 4:**
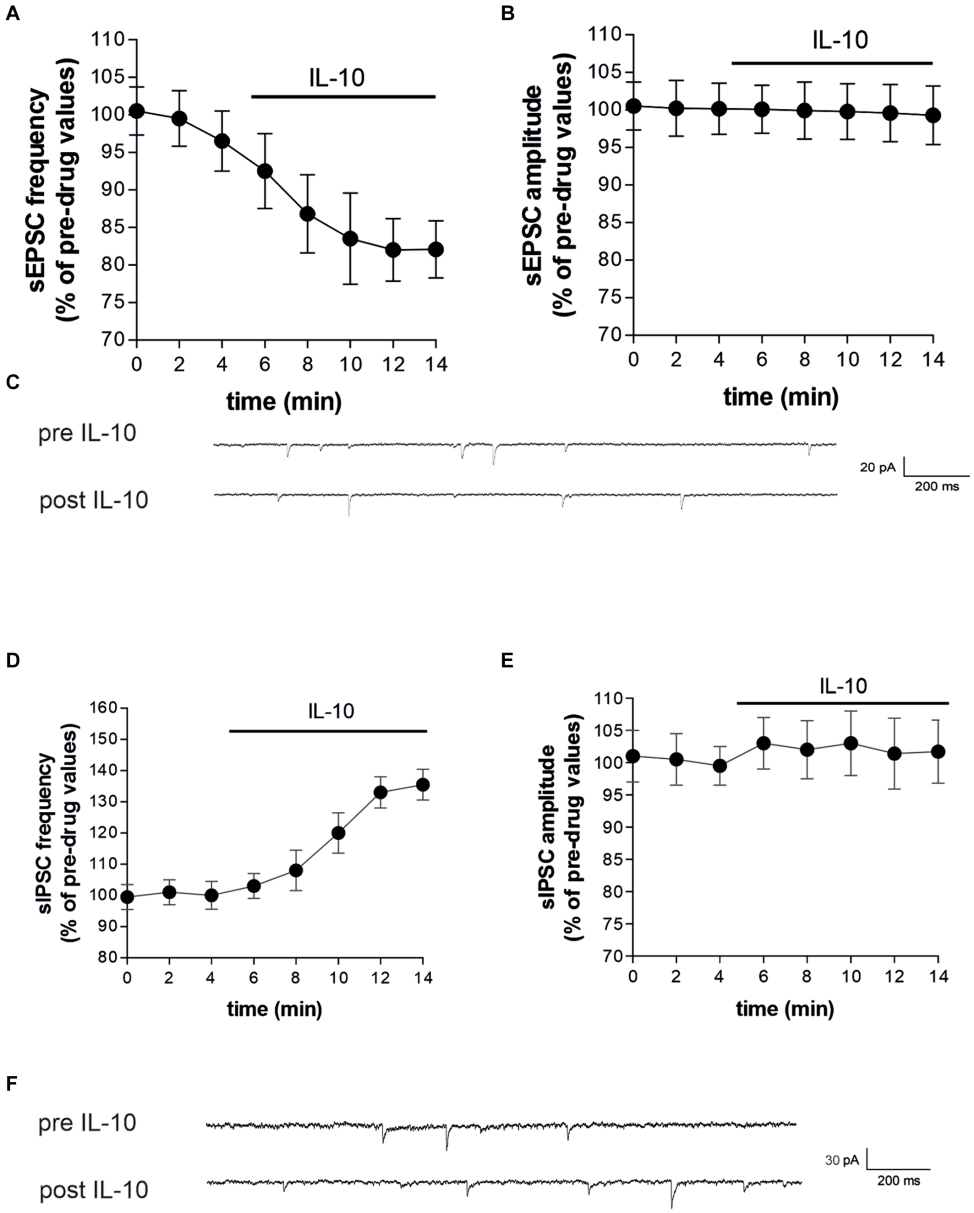
IL-10 incubation reestablished the striatal glutamatergic and GABA-ergic frequencies affected by EAE. **(A–F)** Whole-cell patch-clamp recordings from MSNs of EAE corticostriatal slices incubated with IL-10 (100 ng/mL, 10 min). **(A–C)** The dotted graphs show that IL-10 incubation reduces the frequency **(A)** of EAE sEPSCs with no effect on the amplitude **(B)**, both expressed as percentages of pre-drug values (Paired Student’s *t*-test, *p* < 0.05). **(C)** Representative electrophysiological traces of EAE sEPSCs before and after 10 minutes of IL-10. **(D–F)** The dotted graphs show that IL-10 incubation increases EAE sIPSC frequency **(D)**, with no effect on the amplitude **(E)**, both expressed as percentages of pre-drug values (Paired Student’s *t*-test, *p* < 0.01). **(F)** Representative electrophysiological traces of the EAE sIPSCs before and after ten minutes of IL-10.

The synaptic effect of IL-10 observed in healthy mice suggests a potential anti-excitotoxic effect against IL-1β-induced synaptopathy. In EAE, in fact, the neurodegenerative action of IL-1β was associated with increased frequency and decreased amplitude of, respectively, glutamate-and GABA-mediated synaptic currents by this cytokine (Centonze et al., 2009; Rossi et al., 2011a, 2012a,b; Mandolesi et al., 2015). Thus, to address this hypothesis, we performed electrophysiological recording of EAE corticostriatal slices (20–25 dpi) treated with IL-10. We observed that incubation of IL-10 (7–10 min, 100 ng/mL) reduced the frequency of sEPSC also in EAE mice (Paired *t*-test, *p < 0.05 respect to pre-drug values; *n* = 10; pre-IL-10 = 3.22 ± 0.19–post-IL-10 = 2.66 ± 0.31; Figures 4A,C; Supplementary Figure S3A), showing similar values to control condition (Paired *t*-test, *p* > 0.05; Supplementary Figure S3A). IL-10 failed to modulate sEPSC amplitude (Paired *t*-test, *p* > 0.05; *n* = 10; pre-IL-10 = 12.35 ± 0.64–post-IL-10 = 12.40 ± 0.66; Figures 4B,C; Supplementary Figure S3B), which was also unaffected by EAE (Centonze et al., 2009). In contrast, IL-10 perfusion on EAE corticostriatal slices increased the sIPSC frequencies (Paired *t*-test, **p < 0.01 respect to pre-drug values; *n* = 9; pre-IL-10 = 1.16 ± 0.12–post-IL-10 = 1.68 ± 0.08; Figures 4D,F; Supplementary Figure S3C), counteracting the effects of EAE on inhibitory synaptic transmission (Rossi et al., 2011a), without altering the sIPSC amplitude (Paired *t*-test, *p* > 0.05; *n* = 9; pre-IL-10 = 30.42 ± 1.09–post-IL-10 = 29.92 ± 0.83; Figures 4E,F; Supplementary Figure S3D), supporting the hypothesis of anti-excitotoxic effects promoted by this cytokine. Collectively, these data provided compelling evidence that IL-10 can reduce brain synaptic hyperexcitability in the EAE model.

### 3.6 Il 10 counteracts the IL-1β synaptic effect by acting as a functional antagonist and IL-1 receptor antagonist effects on excitatory synaptic transmission in EAE

In various studies, we investigated the mechanism by which inflammation leads to delayed neuronal damage in multiple sclerosis (Centonze et al., 2009; Haji et al., 2012; Rossi et al., 2012a,b; Gentile et al., 2015; Mandolesi et al., 2015). In one of these studies, we revealed the effect of IL-1β and IL-1ra on cortical excitability in MS patients: the IL-1β/IL-1ra ratio was significantly higher in the CSF of active MS patients and correlated with measures of glutamate transmission in the cortex (Rossi et al., 2012a,b). Furthermore, we demonstrated that co-incubation of CSF from gadolinium-positive (Gd+) MS subjects with IL-1ra normalized the frequency of spontaneous glutamate-mediated excitatory postsynaptic currents (sEPSCs) recorded in brain slices (Rossi et al., 2012a,b).

**Figure 5:**
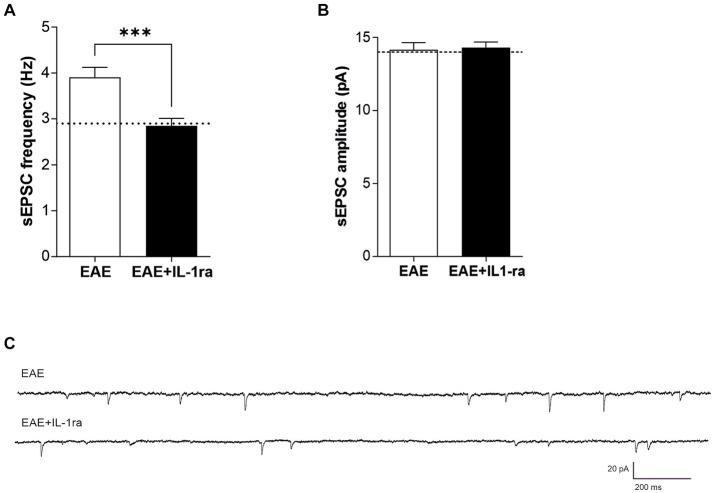
IL-1ra rescues striatal glutamatergic frequencies affected by EAE. **(A–C)** Whole-cell patch-clamp recordings from MSNs of EAE mice pre incubated with IL-1ra (100 ng/mL, 60 min). **(A)** The graphs show that IL-1ra significantly reduced sEPSC frequency of EAE mice (A; Unpaired Student’s *t*-test, *p* < 0.001), without affecting the amplitude (B; Unpaired Student’s *t*-test, *p* > 0.05). **(C)** Representative electrophysiological traces of the sEPSCs of EAE and EAE + ILra.

**Figure 6:**
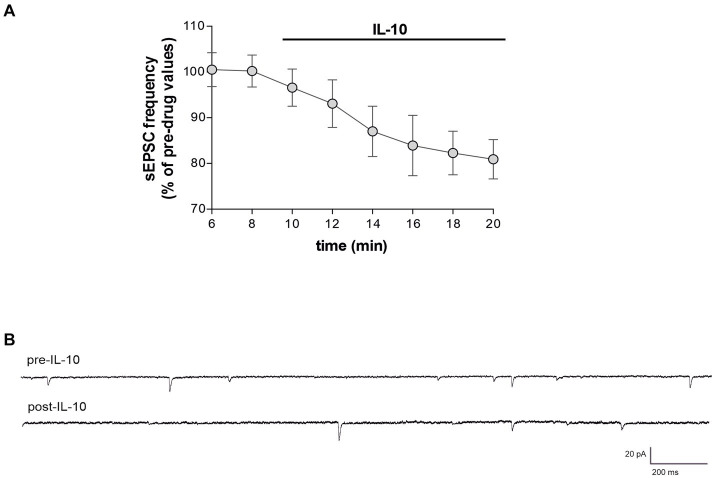
IL-10 counteracts synaptic effects of IL-1 β independently of IL-1β receptor blockade in EAE Slices. **(A)** Whole-cell patch-clamp recordings from MSNs of C57B/L6 corticostriatal slices pre-incubated with IL-1ra (100 ng/mL, 60 min) and with IL-10 (100 ng/mL, 10 min) during electrophysiological recording. **(B)** Representative electrophysiological traces of the sEPSCs before and after 10 minutes of IL-10.

Here we explored for the first time the effect of IL-1ra on glutamate-mediated presynaptic currents of EAE mice. To this aim, EAE slices were-incubated for 1 h with IL-ra (100 ng/mL), and then transferred to a recording chamber. As show in Figures 5A–C, *ex-vivo* treatment is able to recover excitatory frequency of EAE (Unaired *t*-test, ***p < 0.001; EAE *n* = 32 − EAE + IL1ra; *n* = 35), leading to control value (dotted line), demonstrating that IL-1β plays a crucial role in synaptic transmission during EAE, inducing excitotoxic damage. No significant differences were observed on sEPSC amplitude (Unaired *t*-test, *p* > 0.05; Figure 5B). To better understand the mechanism by with IL-10 counteract the synaptic effect of IL-1β, IL-10 was perfused in EAE slices pretreated with IL-1ra. As shown in Figures 6A,B, blocking IL-1 β receptor did not affect the reduction of sEPSCs frequency induced by IL-10 (Paired *t*-test, **p < 0.01; *n* = 8), replicating the observations depicted in Figures 4A–C.

## 4 Discussion

In MS, both white and gray matters are in a subtle balance between inflammation and neuroprotection. The upregulation of genes involved in anti-inflammatory mechanisms may protect the CNS environment and thus limit lesion formation and neuronal damage, whereas the activation of pro-inflammatory mechanisms may favor disease progression. In the present study, by means of clinical and preclinical studies, we propose a synaptic-mediated neuroprotective action of the anti-inflammatory cytokine IL-10 ables to counteract the detrimental action of proinflammatory cytokines, such as IL-1β.

In RR-MS patients, we found a negative correlation between CSF IL-10 levels and clinical disability evaluated one year after the diagnosis. In particular, low IL-10 levels were associated with EDSS worsening, regardless of the possible effect of other clinical characteristics (age at diagnosis, sex, disease duration, radiological activity at diagnosis, EDSS at diagnosis) and DMTs. While this correlation is statistically significant, the strength of the association in relatively weak. Low IL-10 production in stimulated blood lymphocytes was associated with higher disability and MRI lesion load in patients with secondary progressive MS (Petereit et al., 2003). Moreover, we previously reported that increased CSF expression of IL-10 was associated with lower levels of fatigue, reduced white matter lesion load and stable disease course in RR-MS patients (Gilio et al., 2022). Here, we found a positive correlation between CSF IL-10 and the volumes of both caudate and thalamic nuclei evaluated by MRI measurements in a subgroup of RR-MS patients, suggesting an impact of this cytokine also on gray matter damage.

Gray matter pathology plays a documented role in MS progression, in terms of clinical disability and disease course (Cagol et al., 2022). Neuronal atrophy occurs in RR-MS patients since the earliest disease phases (De Stefano et al., 2003; Koubiyr et al., 2018) and has been associated with a proinflammatory CSF milieu (Rossi et al., 2015; Magliozzi et al., 2018, 2020). Studies in experimental models of MS contributed to explain the role of inflammation in triggering neurodegenerative phenomena (Mandolesi et al., 2015). Accordingly, synaptic loss and dysfunction were observed in both presymptomatic and symptomatic phases of EAE in association with infiltrating lymphocytes, microglial activation and upregulation of proinflammatory cytokines (Centonze et al., 2009; Rossi et al., 2011a; Mandolesi et al., 2015; Bellingacci et al., 2021). Chimeric MS *ex-vivo* model obtained by incubating CSF or T-cells derived from MS patients on healthy brain slices, provided further evidence that proinflammatory mediators, such as IL-1β and TNF, are major players of inflammatory synaptopathy in MS (Rossi et al., 2012a,b; Mandolesi et al., 2015).

Our clinical data prompted us to investigate a possible action of IL-10 against inflammatory synaptopathic processes occurring in the gray matter of both MS brain and EAE model. Previous studies reported that anti-inflammatory cytokines may be involved in modulating synaptic excitability and neuronal degeneration in MS brains. For example, high CSF levels of the anti-inflammatory molecule IL-13 were correlated with a more pronounced GABAA-mediated cortical inhibition measured by transcranial magnetic stimulation and a reduced neuronal atrophy evaluated by optical coherence tomography, in line with a possible anti-excitotoxic and neuroprotective effect of this cytokine (Rossi et al., 2011b). Here, we propose that IL-10, which has been previously associated with neuroprotective effects in other experimental neurological conditions, counteracts inflammatory synaptopathy by attenuating synaptic hyperexcitability. We first explored the physiological effect of IL-10 on striatal transmission in control mice, and found that this cytokine can reduce glutamatergic transmission and increase GABAergic activity. We also investigated the effects of IL-10 on synaptic transmission during neuroinflammation, showing that this cytokine normalized both glutamatergic and GABAergic transmission when directly perfused on EAE striatal slices. Altogether these results suggest a pivotal role of IL-10 in controlling excitotoxic imbalance, limiting the neurodegenerative cascade in the basal ganglia.

Our previous finding showed that IL-1β was able to increase glutamatergic synaptic transmission and to inhibit GABAergic inhibitory postsynaptic currents in MS chimeric *ex-vivo* models (Rossi et al., 2012a,b). Accordingly, we demonstrated that blockade of IL-1β activity prevents synaptic hyperexcitability mediated by CSF from gadolinium-positive (Gd1) MS subjects and improves the clinical course of EAE mice (Rossi et al., 2012b; Mandolesi et al., 2013) and that disability progression and excitotoxic-degenerative processes in RR-MS patients likely depends on an enhancement of IL-1β signaling and related regulatory axis (Rossi et al., 2012a,b; Rossi et al., 2014; De Vito et al., 2022). We therefore suggest that, during an acute attack of MS, inflammation increases brain IL-1β signaling, which enhances in turn neuronal excitability and neurotoxicity (Rossi et al., 2012a,b).

In this study we demonstrated for the first time that IL-1ra is able to rescues glutamate release also in EAE slices, indicating a role of IL-1β on striatal excitotoxicity in MOG-immunized mice.

IL-10 appears to play a crucial role in counteracting excitotoxicity due to excessive stimulation by neurotransmitters such as glutamate, and the mechanism by which IL-10 exerts its protective effects seems to involve a functional antagonism that mitigates excitotoxic damage. IL-1β is often associated with promoting inflammation and contributing to excitotoxic damage in the brain (Mendiola and Cardona 2018). Here, by sequentially applying IL-1ra and IL-10 to the same striatal slice we demonstrated that the beneficial effects of IL-10 occur independently of IL-1β signaling. The independence from IL-1 β signaling suggests that IL-10 might be engaging different molecular pathways to exert its neuroprotective effects. Further research into the precise mechanisms and pathways involved could provide deeper insights into how IL-10 can be harnessed for clinical applications.

IL-10 is one of the most studied regulatory cytokines associated with anti-inflammatory activity. It is able to decrease immune cell activation and production of pro-inflammatory mediators (Kwilasz et al., 2015). Studies in animal models have indicated a role of IL-10 in the pathogenesis of MS. Over the past decades research has unveiled protective effects of IL-10 on EAE through genetic and pharmacological approaches. EAE mice lacking IL-10 showed progressive clinical deterioration accompanied by pronounced immune cell activation and inflammation (Bettelli et al., 1998; Samoilova et al., 1998a; Anderson et al., 2004). IL-10-deficient mice (IL-10 −/−) were highly susceptible to severe EAE compared to wild-type (WT) controls, with IL-10 −/− mice developing chronic disease without remission while WT mice experienced complete disease remission (Samoilova et al., 1998b). Conversely, increased levels of this cytokine reduced brain inflammation and neurodegeneration (Cua et al., 2001; Strle et al., 2001; Waubant et al., 2001; Gilio et al., 2022), and prevented EAE induction in IL-10 transgenic mice (Bettelli et al., 1998; Cua et al., 1999).

B cell regulatory function is primarily mediated by IL-10 secretion (Matsumoto et al., 2014). It has demonstrated suppression of various autoimmune and inflammatory diseases like arthritis, inflammatory bowel disease, and contact hypersensitivity (Keystone et al., 1998; Li and He 2004; Girard-Madoux et al., 2012). Notably, B cell-produced IL-10 has been shown to mitigate EAE severity (Fillatreau et al., 2002), playing a crucial role in preserving blood–brain barrier (BBB) integrity (Dai et al., 2012). Studies indicate IL-10 −/− mice exhibited typical clinical EAE progression, unchanged CNS inflammation, and comparable BBB damage, underscoring necessity of IL-10 for beneficial effects in EAE (Dai et al., 2012). These results highlight the potential of IL-10 as a therapeutic target for modulating immune responses in CNS-related autoimmune disorders like EAE.

Based on the findings of Croxford et al. (2001), local CNS delivery of IL-10 through retroviral gene therapy demonstrated significant therapeutic benefits in EAE. This treatment not only ameliorated EAE symptoms but also arrested disease progression. The observed increase in the frequency of CD8+ T cells and B cells in IL-10-treated mice suggests that IL-10 promotes the proliferation of activated CD8+ cells (Croxford et al., 2001). Furthermore, a recent study published in Nature Communications underscored IL-10’s therapeutic effect on EAE through B cells. By manipulating SLAMF5, a negative regulator of Breg levels, researchers found that SLAMF5-deficient mice had delayed EAE onset, less severe disease, and lower incidence rates compared to WT mice (Radomir et al., 2021).

In contrast with these results, in a study of Cannella et al., researchers found that IL-10 is insufficient to reverse the effector response and may actually exacerbate the cascade of events in EAE. In particular, when IL-10 was administered for six days before the onset of symptoms, no significant amelioration was observed. During the chronic phase of EAE, IL-10 administration showed no difference in inflammation between the IL-10 and PBS-treated groups; however, demyelination was more prominent in the IL-10 treated animals. Otherwise, treatment with IL-10 antibodies prior to the acute phase of EAE resulted in more severe pathology in the treated group (Cannella et al., 1996).

In addition to its anti-inflammatory effects, IL-10 can modulate neuronal function by up-regulating the expression of pro-neural genes during postnatal neurogenesis or pro-survival genes after injury (Zhou et al., 2009; Pereira et al., 2015). Interestingly, different experimental data agree that IL-10 exerts a neuroprotective effect by modulating aberrant neuronal activity associated with inflammatory mediators. In a study focused on neuropathic pain, IL-10 was able to reduce dorsal root ganglion (DRG) neuron excitability by contrasting the enhancement of voltage-gated sodium current mediated by TNF (Shen et al., 2013). In the hippocampus, IL-10 can occlude negative effects of IL-1β on long-term plasticity and basal synaptic transmission, likely by favoring the shedding of the IL-1 type I receptor (Kelly et al., 2001; Nenov et al., 2019). Furthermore, increased hippocampal levels of IL-10 were associated with reduced excitability of the CA3-CA1 synapses and with loss of long-term potentiation (Almolda et al., 2015). In addition, a role of IL-10 in contrasting glutamate-mediated neuronal apoptosis has been reported (Bachis et al., 2001; Zhou et al., 2009) and in IL-10 knockout mice, cortical neurons were more vulnerable to excitotoxic damage (Grilli et al., 2000).

## 5 Conclusion

Our data provide further evidence that IL-10 plays a crucial role in synaptic homeostasis and neuronal survival, and indicate that IL-10 exerts direct neuroprotective effects in MS patients and of EAE mice, through the modulation of both excitatory and inhibitory transmission and the mitigation of inflammatory synaptopathy induced by IL-1β.

## Data availability statement

The datasets presented in this study can be found in online repositories. The names of the repository/repositories and accession number(s) can be found at: https://repository.neuromed.it/index.php/login, NT_010524.

## Ethics statement

The studies involving humans were approved by Ethics Committee of IRCCS Neuromed (CE numbers 06/17). The studies were conducted in accordance with the local legislation and institutional requirements. The participants provided their written informed consent to participate in this study. The animal study was approved by Organismo Preposto al Benessere Animale (OPBA) del Centro di Servizi Inerdipartimentale, Stazione per la tecnologia Animale, Università degli Studi di Roma Tor Vergata—D. Lgs. 26/2014. The study was conducted in accordance with the local legislation and institutional requirements.

## Author contributions

LuG: Conceptualization, Data curation, Project administration, Supervision, Validation, Visualization, Writing – original draft. DF: Conceptualization, Project administration, Validation, Data curation, Investigation, Writing – original draft. MSB: Data curation, Formal analysis, Funding acquisition, Investigation, Writing – original draft. AM: Formal analysis, Investigation, Writing – original draft. FDV: Funding acquisition, Investigation, Writing – review & editing. SB: Data curation, Writing – review & editing. KS: Investigation, Writing – review & editing. SC: Investigation, Writing – review & editing. LP: Investigation, Writing – review & editing. GG: Data curation, Investigation, Writing – review & editing. IS: Formal analysis, Writing – original draft, Writing – review & editing. LiG: Investigation, Writing – review & editing. VV: Data curation, Writing – review & editing. FB: Funding acquisition, Investigation, Writing – review & editing. ED: Investigation, Writing – review & editing. ABr: Investigation, Writing – review & editing. FA: Data curation, Writing – review & editing. ABo: Investigation, Writing – review & editing. RFa: Investigation, Writing – review & editing. AF: Investigation, Writing – review & editing. RFu: Investigation, Writing – review & editing. DC: Conceptualization, Funding acquisition, Project administration, Resources, Supervision, Validation, Visualization, Writing – review & editing. GM: Conceptualization, Data curation, Funding acquisition, Methodology, Project administration, Resources, Supervision, Validation, Visualization, Writing – review & editing.
