# Supplementary material for: Interleukin-10 contrasts inflammatory synaptopathy and central neurodegenerative damage in multiple sclerosis
Source: Front Mol Neurosci. 2024 Aug 7;17:1430080. doi: 10.3389/fnmol.2024.1430080 (PMC11338018; doi:10.3389/fnmol.2024.1430080)
Supplement: Supplementary file 1 [file Data_Sheet_1.docx]

**Supplementary Figure 1.**

**Representative segmentations and labels of subcortical regions of interest (ROIs). The segmentations are overlaid on the T1-weighted magnetic resonance scan.**


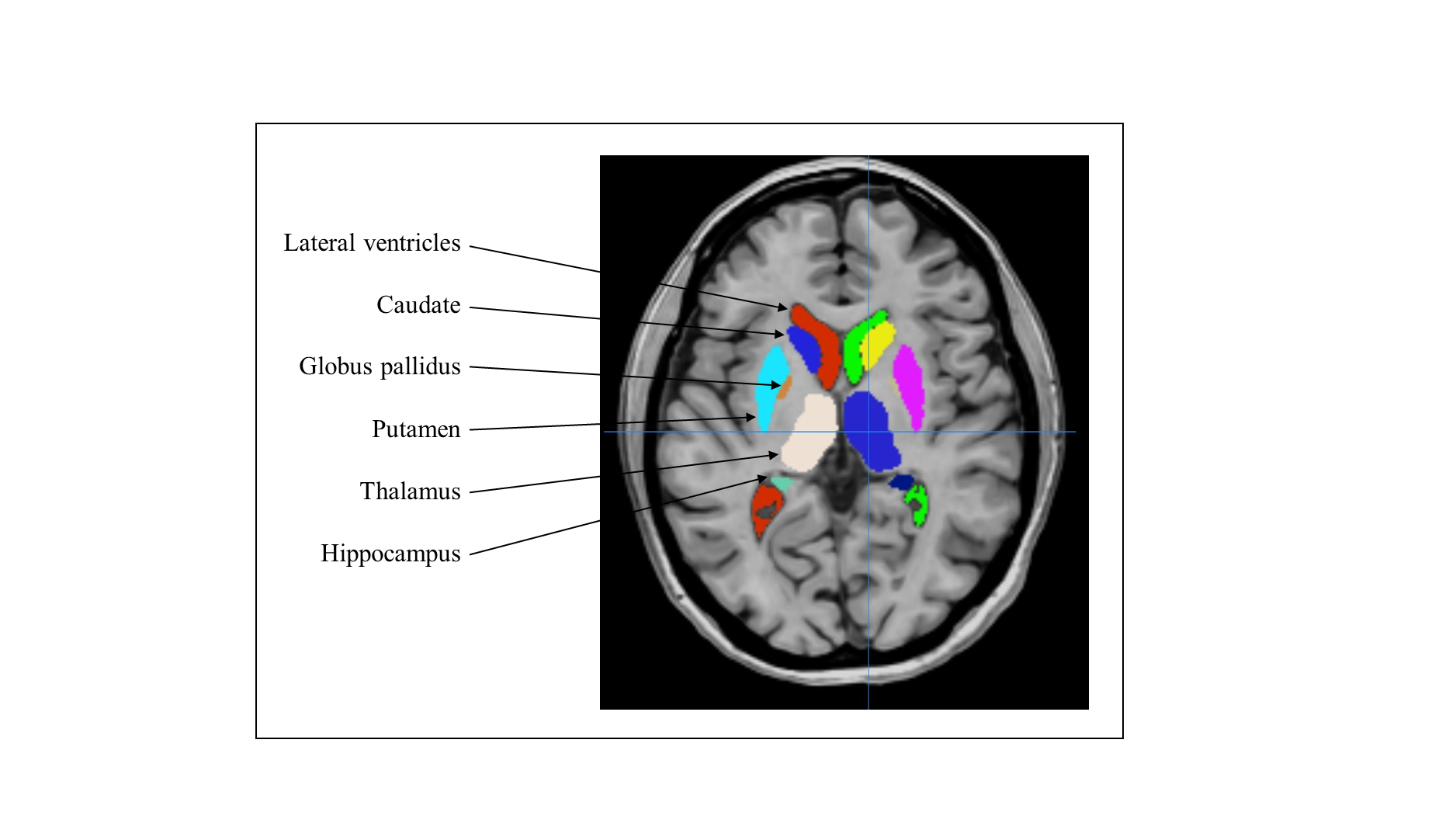


**Supplementary Figure 1 legend.**

All ROIs were segmented automatically (see [Methods](https://www.sciencedirect.com/science/article/pii/S1353802013003052#sec2)).
