# Supplementary material for: Interleukin-10 contrasts inflammatory synaptopathy and central neurodegenerative damage in multiple sclerosis
Source: Front Mol Neurosci. 2024 Aug 7;17:1430080. doi: 10.3389/fnmol.2024.1430080 (PMC11338018; doi:10.3389/fnmol.2024.1430080)
Supplement: Supplementary file 2 [file Table_1.docx]

**Supplementary Table 1.** **Effect of IL-10 on the EDSS variation**

|  | **Beta** | **SE** | **p** |
| --- | --- | --- | --- |
| Age at diagnosis | 0.01 | 0.004 | 0.059 |
| Sex, F | -0.001 | 0.098 | 0.994 |
| Disease duration, months | 0.002 | 0.001 | 0.104 |
| Radiological activity at diagnosis | -0.09 | 0.095 | 0.363 |
| DMT type, II line | -0.02 | 0.111 | 0.846 |
| CSF IL-10 (log scale) | -0.76 | 0.23 | 0.001 |
| EDSS at diagnosis | -0.23 | 0.045 | <0.001 |

**Supplementary Table 1 legend.**

Regression model adjusting for age at diagnosis, sex, disease duration, radiological activity, EDSS at diagnosis, and DMT type (I vs II line).
